# Rapamycin inhibits oral cancer cell growth by promoting oxidative stress and suppressing ERK1/2, NF-κB and beta-catenin pathways

**DOI:** 10.3389/fonc.2022.873447

**Published:** 2022-09-15

**Authors:** Abdelhabib Semlali, Sofia Papadakos, Camille Contant, Ikram Zouaoui, Mahmoud Rouabhia

**Affiliations:** Groupe de recherche en écologie buccale, Faculté de médecine dentaire, Université Laval, Québec, QC, Canada

**Keywords:** rapamycin, oral cancer, apoptosis, autophagy, oxidative stress, MAPK, Wnt pathway

## Abstract

Treatment of oral cancer is based exclusively on surgery combined with or without chemotherapy. However, it has several side effects. Targeting a new, more effective therapy has become an urgent matter. The purpose of this study was to evaluate the anti-tumor activity of rapamycin in oral cancer and its mechanism of action. Human gingival carcinoma cells were stimulated with different concentrations of rapamycin to assess proliferation, colony formation, cell migration, as well as apoptosis, and autophagy. The expression of proteins involved in the cell cycle (cyclin D1, p15, p21, p27) and autophagy, as well as that of oncogenes and tumor suppressor genes, were determined by quantitative PCR. The signaling pathways were evaluated by Western blotting. Our results show that rapamycin has a selective effect at a low dose on cancer cell growth/survival. This was confirmed by low colony formation and the inhibition of cell migration, while increasing cell apoptosis by activating caspase-9 and -3. Rapamycin promoted cell autophagy and increased mitochondrial oxidative stress by being involved in DNA damage in the exposed cells. Finally, rapamycin exhibits potent anti-oral cancer properties through inhibition of several cancer-promoting pathways (MAPK, NF-κB, and Wnt/beta-catenin). These results indicate that rapamycin could be a potential agent for the treatment of oral cancer and for a prevention strategy.

## Introduction

Oral cancer is the sixth most malignant disease in the world (1). It can be caused by an alteration in epigenetic, genetic, or environmental factors (1). The conventional treatment for cancer is a combination of surgery with radiation therapy or chemotherapy (1). But this has several adverse effects such as fatigue, vomiting, appetite loss and pain, which may vary for each person in terms of severity (2). Cisplatin is the drug used for chemotherapy treatment as it is the most effective agent against cancer (3). However, this molecule causes negative effects as it tags cancerous and healthy cells (2). Therefore, it is urgent to develop a new therapeutic strategy against oral cancer. Several studies have sought alternative treatments by targeting the inhibition of many signaling pathways in cancer. The mechanistic target of rapamycin (mTOR) is an evolutionary conserved serine-threonine kinase present in multiple cancers. This protein kinase controls a wide variety of cellular functions involved in cell growth and proliferation (4, 5). Rapamycin has antimicrobial and immunosuppressive properties (1; 6–8). More recently, rapamycin was considered as an anti-cancer molecule (9). The mTOR signaling pathway often becomes poorly activated during tumor progression and contributes to tumorigenesis by deregulating cancer cell proliferation. By targeting mTOR signaling, rapamycin shows promise for inhibiting the growth of tumors (1). Rapamycin inhibits cancer cell growth, inducing cancer cell apoptosis, and suppressing tumor angiogenesis (10). This anti-tumor property of rapamycin has been studied with endometrial (11) and breast cancer (12). However, no studies have been made regarding rapamycin and oral cancer. The objective of this study was to *in vitro* evaluate the effect of rapamycin on human gingival carcinoma cell proliferation, apoptosis, and autophagy.

## Methods and materials

### Cells

The human gingival epithelial carcinoma cell line Ca9-22 was purchased from RIKEN BioResource Research Center (Tsukuba, Japan). The culture of the Ca9-22 was in RPMI-1640 supplemented with 5% fetal bovine serum (FBS). We also included in this study human polyclonal oral epithelial cell line (GMSM-K) provided by Dr. Grenier (Université Laval). The GMSM-K cell line was constructed by Gilchrist et al. (2000) who transfected oral epithelial cells with the shuttle vector plasmid, pZ189, containing the T Antigen Coding Region and Replication Origin from the simian virus 40 (SV40). This cell line was grown in Dulbecco’s Modified Eagle’s Medium (DMEM) supplemented with 5% FBS and 1% penicillin-streptomycin solution (Sigma Aldrich, St. Louis, MO, USA) both cell lines were maintained in culture at 37°C in a 5% CO_2_ atmosphere.

### Reagents

Rapamycin was purchased from MedChemExpress LLC (NJ 08540, USA). MTT, protease and phosphatase inhibitors were from Sigma-Aldrich (Oakville, Ontario, Canada). LDH-Cytotoxicity Colorimetric Assay Kit II was purchased from BioVision (Milpitas, California, USA), while Annexin V-FITC/PI Kit was from BD Bioscience. (Mississauga, ON, Canada). Autophagy Assay, Red (Cat. #9156), Intracellular Total ROS Activity Assay (Cat. #9144) and Intracellular GSH Assay (Cat. #9137) were purchased from ImmunoChemistry Technologies (Davis, CA, USA). Autophagy Inhibitor, 3-MA (Cat. #189490) and N-acetylcysteine (NAC) were from Sigma. ECL system was acquired from EMD Millipore (Billerica, MA, USA). The primary antibodies as procaspase 3 (sc-56046), procaspase 9 (sc-17784), NF-κB (sc-8008) and β-catenin (sc-59737) were purchased from Santa Cruz Biotechnology (Santa Cruz, CA, USA), E-cadherin (8834), pERK1/2 (4370), ERK1/2 (4695), pp38 (4631), p38 (9212), cleaved caspase-3 (9664S), cleaved caspase-9 (20750S) were from Cell Signaling Technology (Danvers, MA, USA), LC3B (2775) and p62 (39,749) were all from Cell Signaling Technology (Danvers, MA, USA)and β-actin (A5441) was from Sigma-Aldrich (Oakville, ON, Canada). The secondary goat anti-mouse (554002) and anti-rabbit (554021) were from BD Pharmingen (Mississauga, ON, Canada). VersaDoc™ MP 5000 system was from Bio-Rad (Mississauga, ON, Canada).

### Cell viability assay and nucleus staining

Cell proliferation was evaluated using MTT assay as well as nucleus staining. Briefly, Ca9-22 and GMSM-K cells were seeded into 12-well plates at the density of 3 x 10^5^ cells/well, cultured overnight and then exposed to different concentrations of rapamycin (from 0.1 to 100 µM) for 24 h. After incubation, the culture medium was replaced with a new one containing MTT solution of 5 mg/ml in PBS for 3 h at 37°C in the dark, as described by Semlali and al. previously (13, 14). The cells were incubated for 15 min in 1 ml HCl 0.05 N-isopropanol solution to lyse the cells and release the formed formazan. The solution was transferred to 96-well microplate with 200 µl per well, and the absorbance was measured at 550 nm by an iMark reader (Bio-Rad). Percentage of viable proliferating cells was determined by using the following formula: % of cell viability = [(OD_550 nm_ (treated cell) − OD (blank)/(OD (control cell) − OD (blank))] × 100. The IC50 of rapamycin was obtained by plotting the percentage inhibition of cell proliferation against rapamycin concentration. The experiment was repeated eight times.

For nucleus staining, oral cancer cells (Ca9-22) at 10^5^ were seeded into sterile glass slides immersed in RPMI-1640 medium supplemented or not with different concentrations of rapamycin (0, 0.1, 1, 10, or 20 and 100 μM). The cells were cultured for 24 h at 37°C in a 5% CO_2_ incubator. After 24h the cells were fixed with 4% paraformaldehyde for 60 min at room temperature before Hoechst staining (10 μg/ml) for 15 min. Finally, the slides were washed three times with the phosphate buffered saline (PBS), observed under an epifluorescence microscope (Nikon Optiphot) and photographed with a digital camera (Nikon COOLPIX 995). The experiment was repeated three times.

### Cell cytotoxicity by LDH assay

Cellular toxicity was determined by the LDH-Cytotoxicity Detection Kit from BioVision, which allows to directly quantify cell death in culture, based on the measurement of lactate dehydrogenase (LDH) released into growth media (15). Briefly, 3.10^5^ cells per well were to be seeded into six-well plates and incubated for 24h, before being exposed to different concentrations of rapamycin for an extra 24 h. Afterward, 50 μl of each supernatant was transferred in triplicates to a 96-well plate and supplemented with 50 μl reconstituted substrate mix. Then, the plates were incubated for 30 min at room temperature in the dark until the yellow color developed, before reading at 490 nm with a xMark microplate absorbance spectrophotometer (Bio-Rad, Mississauga, ON, Canada). Triton X-100 (1%) was used as a positive control for LDH and the negative one was obtained with untreated cells. LDH release was calculated using the following formula: % of LDH activity = [rapamycin (absorbance) − negative control (absorbance)] × 100)/[positive control (absorbance) − negative control (absorbance)]. The experiment was repeated four times.

### Clonogenic assay

Ca9-22 cells were seeded into 6-well plates at the density of 2000 cells per well for 24 h. They were then stimulated with different concentrations of rapamycin, ranging from 0 to 100 μM. The cells were incubated for two weeks at 37°C in 5% CO_2._ The culture medium was changed every 2 or 3 days. The colonies were fixed with 100% ethanol and then stained with 0.5% crystal violet solution, as described by our previous works (13, 14). The colonies were subsequently washed twice with deionized water, dried at room temperature, observed under an optical microscope, and finally photographed. The experiment was repeated three times.

### Cell apoptosis detected by flow cytometry with Annexin/PI protocol

The cells were cultured and stimulated with different concentrations of rapamycin for 24 h at 37°C and 5% CO_2_. They were then detached with a solution of 0.05% trypsin and 0.01% EDTA, incubated with Annexin V-FITC and propidium iodide at room temperature for 30 min in the dark. Finally, cells were resuspended in 300 µl of the phosphate buffered saline (PBS), to perform a flow cytometry, using either BD LSR II or BD FACSCanto II cytometer (BD Bioscience) equipped with FACSDiva Software v. 6.1.3. The experiment was repeated four times.

### Wound-healing assay

Cell migration assay was performed as described previously by Semlali and al. (13, 14). Ca9-22 cells were seeded into 6-well plates and cultured until they reached 100% confluence. Cell monolayers were subjected to a scratch in the shape of a cross with a sterile pipette tip. The cells were then stimulated with different concentrations of rapamycin, ranging from 0 to 100 μM and incubated at 37°C in a humid atmosphere containing 5% CO_2_. Photographs were taken of each well with an inverted microscope after 0, 6, 12 and 24 h after the scratch was made. The cell migration was analyzed by image processing software that was able to measure the distance between opposite edges of the scratch at each time point. Each well was then compared, based on their percentage of closure. The experiment was repeated three times.

### Quantification of cellular autophagy

To evaluate the effect of rapamycin on autophagy in Ca9-22 cells, we used flow cytometry analyses as described previously (13, 14). Briefly, Ca9-22 cells were seeded into 60 mm Petri dishes for adhesion overnight. Afterward, cells were treated with controls of vehicle alone (0.2% of DMSO) or with 10 and 20 μM of rapamycin for 24 h. Following the rapamycin treatment, cells were resuspended in 500 μl of culture medium containing 1/5 Red staining solution. Cells were incubated for 60 minutes at 37°C in the dark and then collected by low centrifugation. The cell pellet was washed with 500 μl of the 1X Assay Buffer three times and suspended in 500 μl fresh 1X Assay Buffer before analyzing it with the green (FL1) channel of a flow cytometer using BD LSR II or BD FACSCanto II system (BD Bioscience) equipped with FACSDiva Software v. 6.1.3. The experiment was repeated four times.

### Determination of ROS levels by flow cytometry

Oxidative stress was assessed by flow cytometry using ROS marker protocol from ImmunoChemistry Technologies. After stimulation with rapamycin at (0, 10 and 20 μM) for 24 h, cells were detached with trypsin, washed with PBS, and were then resuspended in 490 μl of culture medium supplemented with 10 μl Green ROS stain solution and incubated in the dark for 1 h at 37°C. Fluorescent intensity of labeled cells was analyzed by flow cytometry at 488 nm using BD LSR II or BD FACSCanto II cytometer (BD Bioscience). The percentage of positive results was calculated in living cells with FACSDiva Software v. 6.1.3. This experiment was repeated four times.

### Measurement of mitochondrial superoxide

Generation of mitochondria-mediated ROS was evaluated by using the MitoSOX-Red Mitochondrial Superoxide Indicator (Invitrogen). Firstly, Ca9-22 cells were treated with different concentrations of rapamycin (10 and 20 µM) for 24h. Subsequently, cells were harvested, washed twice in PBS and incubated with 5 mmol/l of mitochondrial dye (MitoSOX Red; Molecular Probes, Invitrogen) for 30 min at 37°C in the dark, followed by analysis on a flow cytometer to calculate the percentage of MitoSox-positive cells. This experiment was repeated four times.

### Assessment of DNA damage by flow cytometry

As described by our previous studies (13, 14, 16), to evaluate the effect of rapamycin on damage to oral cancer cells, a H2A.X flow cytometry was performed. In addition, after treating Ca9-22 cells with the studied concentrations of rapamycin, they were trypsinized and then fixed with 75% ethanol for 15 min. The centrifugation of the samples was carried out to eliminate the fixative solution. Afterward, a permeabilization solution containing 1% BSA/0.2% Triton/1X PBS was added to the cells and they were then incubated in the dark at 4°C overnight with the first phospho-histone H2A.X (Ser139) monoclonal antibody from Santa Cruz Biotechnology at a dilution 1/100, washed twice with PBS and incubated with the secondary antibody conjugated to Alexa Fluor 488 from Santa Cruz Biotechnology in a 1:100 ratio for 1h before analyzing them with the BD flow cytometry system (BD FACS Canto II) and the percentage of positive cells was calculated. This experiment was repeated three times.

### Real-time reverse transcription PCR (qPCR) analysis for gene expression studies

Total RNA was extracted from treated and untreated cells by using the RNeasy Mini Kit from Qiagen (Toronto, Ontario, Canada). An amount of 1 μg total RNA was reverse-transcribed into a cDNA copy with the High-Capacity cDNA Reverse Transcription Kit from Applied Biosystems (Thermo Fisher Scientific, USA) according to the manufacturer’s instructions, as described by our previous works (17–19). The RNA concentration and purity were determined by using a Nanodrop 8000 spectrophotometer (Thermo Fisher Scientific, USA). The qPCR protocol was performed with a 7500 Real-Time PCR System (Applied Biosystems). The reaction volume for each sample consisted of 12.5 µl SYBR Green Master Mix 2X, 0.5 µl primer (Forward and Reverse) (See **Table 1**), 7 µl distilled water, and 5 µl cDNA. The conditions for the PCR hold were 95°C for 5 min, followed by 40 cycles at 95°C for 15 sec, 60°C for 30 minutes and 30 sec at 72°C. The results were then analyzed using the Livak method for relative expression. The experiment was repeated three times.

**Table 1:** Primer sequences used for the qRT-PCR.

Gene	Primer sequences	Product length size (pb)
Cyclin D1	**F : 5'-**AGCTGTGCATCTACACCGAC-**3'** **R : 5'**GAAATCGTGCGGGGTCATTG-**3'**	**113**
p21 (**CDKN1A**)	**F :5'-**TGCCGAAGTCAGTTCCTTGT-**3'** **R :5'**CATTAGCGCATCACAGTCGC-**3'**	**190**
p15 ( **CDKN2B** )	**F :5'-**TTTACGGCCAACGGTGGATT-**3'** **R:5'**CATCATCATGACCTGGATCGC-**3'**	**220**
p27	**F** :**5’**- TTGCGCAATTAGGTTTTTCC-**3’** **R** **:5’**-AAAGGAATTCAAGCCTTCC-**3’**	**64**
LC3B	**F :5'-**TCAGGTTCACAAAACCCGCC-**3'** **R :5'**GCGTTTGTGCCAACTGTGAT-**3'**	**140**
p62	**F :5'-**GCCATTGCGGAGCCTCATCT-**3'** **R :5'**CAGCCATCGCAGATCACATTG-**3'**	**322**
GAPDH	**F :5'-**GGTATCGTGGAAGGACTCATGAC-**3'** **R :5'**ATGCCAGTGAGCTTCCCGTTCAGC-**3'**	**188**

### Western blot

Ca9-22 cells at the density of 10^6^ were harvested for extraction by a lysis buffer. A Bradford protein assay was then conducted to determine the protein concentrations of each sample. An amount varying between 20 μg and 60 μg of proteins were separated by SDS-PAGE with 8–15% of acrylamide, electro transferred onto nitrocellulose membrane. The membrane was blocked in a 5% milk solution at room temperature for 1 h and then incubated overnight with the specific primary antibodies. The membrane was then left in the secondary antibody solution for 1 h before being rinsed four times with washing solution. Detection of proteins was carried out with an enhanced chemiluminescence (ECL) Western Blotting Kit according to the manufacturer’s instructions and revelation by Versa Doc™ MP 5000 system (Bio-Rad, Mississauga, ON, Canada).

### Statistical analysis

The significant difference between experimental (treated) groups and controls (untreated) was evaluated by Student’s *t-*test in GraphPad Prism 7 Software. Error bar represented mean ± SEM. ^*^ *P-* value < 0.05 was statistically significant.

## Results

### Rapamycin at a low dose selectively inhibits the proliferation of Ca9-22 cells

**Figure 1:**
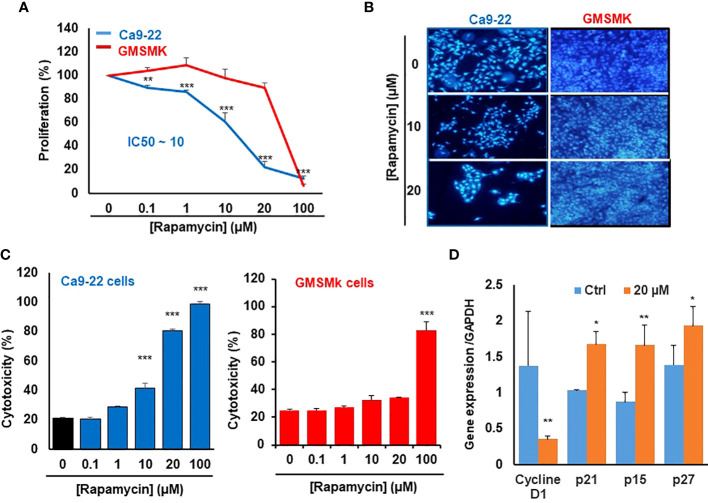
Rapamycin at a low dose selectively inhibits the proliferation of Ca9-22 cells. **(A)** Cell growth was measured by MTT assay. Ca9-22 cells were stimulated by rapamycin concentration, from 0.1 to 100 µM for 24h. Results are expressed as mean percentage of proliferation ± SD. The untreated cells represent 100% of proliferation. (n = 8 for Ca9-22 and n = 6 for GMSM-K cells). **(B)** Nucleus staining, Ca9-22 and GMSM-K cells were seeded into sterile glass slides immersed in culture medium. After 24h of rapamycin treatment, cells were fixed with 4% paraformaldehyde before Hoechst staining (10 μg/ml). The experiment was repeated three times. **(C)** Cytotoxicity was assessed by LDH assay. Cells were stimulated by the same rapamycin concentrations for 24h. The LDH activity was presented as mean percentage of cytotoxicity ± SD. *P*-value was considered as significant when it was < 0.05 (comparison between untreated and rapamycin-treated cells). **(D)** Rapamycin inhibits proliferation by blocking the cell cycle in Ca9-22 cells. They were treated with 20 µM of rapamycin for 24h. After, total RNA was extracted, and reverse-transcribed into a cDNA. By using qPCR, we evaluated the expression of cell-cycle inhibitors such as p21, p15, p27 and cyclin D1 (n = 3). *P < 0.05, **p < 0.005, or ***p < 0.0005.

We first investigate the effect of rapamycin on the proliferation of human gingival epithelial carcinoma cells (Ca9-22) and human polyclonal oral epithelial cells (GMSM-K). As shown in **Figure 1A**, rapamycin inhibited the proliferation of Ca9-22 cells in a dose-dependent manner. The half-maximal inhibitory concentration (IC_50_) value was around 15 μM of rapamycin (**Figure 1A**). In addition, only the high concentration of rapamycin (100 µM) affects the proliferation of normal gingival epithelial cells (GMSM-K). These results were confirmed with the nucleus staining assay. **Figure 1B** shows that the number of nuclei in cancer cells dramatically decreases in the presence of rapamycin, while no effect was seen with GMSM-K cells (**Figure 1B**). The anti-proliferation effect of rapamycin evaluated by MTT was confirmed by LDH assay. As shown in **Figure 1C**, rapamycin increased the LDH activity of Ca9-22 cells in a dose-dependent manner. The median inhibitory concentration was also calculated and was around 15 µM. For GMSM-K, rapamycin induced cell toxicity only for 100 µM; the same effect was observed by MTT assay for this cell type (**Figure 1C**). These results show that a high dose of rapamycin non-selectively inhibits the viability of human oral cancer and normal cells. However, when the cells were treated with lower concentrations of rapamycin (non-cytotoxic to normal oral cells), we witnessed a significant inhibitory effect on oral cancer cell proliferation. We next investigated whether rapamycin affected the cell morphology.

Differences in cell morphology were observed between rapamycin-treated oral cancer cells and controls (treated with DMSO) under light microscopy (Data not shown), the most dramatic morphology changes were seen with rapamycin-treated cells at low concentrations of rapamycin (10, 20 µM) and high concentration (100 µM) in Ca9-22 cells, but only at 100 µM for normal oral cells. These morphology changes are manifested by cell shrinkage, cells became round and lost their integrity to promote cancer progression as well as their number was significantly reduced, and there was an extensive detachment from the cell culture substratum compared with the vehicle. The morphology changes observed in Ca9-22 cells are probably characteristics of oral cancer cell death and apoptosis.

The mechanism by which rapamycin inhibits oral cancer cell proliferation is probably through the cell cycle. This was confirmed by studying the effect of rapamycin on cell-cycle inhibitors. As shown in **Figure 1D**, rapamycin at 20 µM (a concentration close to the IC_50_ value) was inhibiting cell proliferation by induction of cell-cycle inhibitors such as p21, p15 and p27 and the repression of cyclin D1 expression. Therefore, rapamycin inhibits proliferation by blocking the cell cycle.

### Rapamycin selectively suppresses oral cancer cell growth/survival by inhibition of colony-forming cells

**Figure 2:**
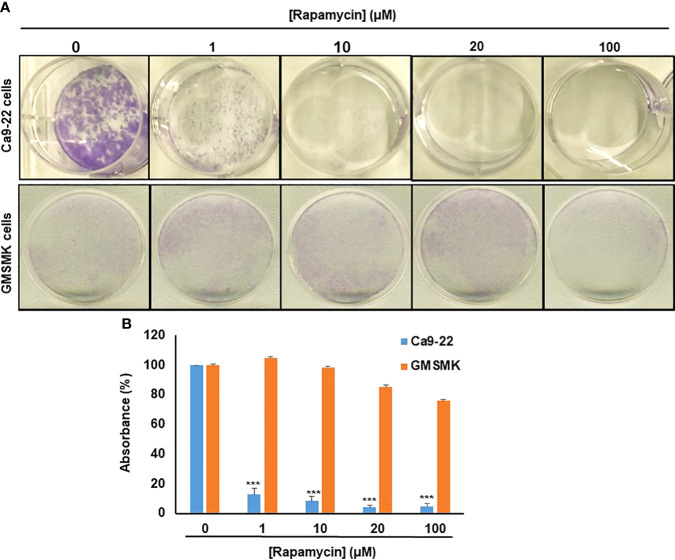
Rapamycin selectively suppresses oral cancer cell growth/survival by inhibition of colony-forming cells. **(A)** Colony formation of Ca9-22 and GMSM-K cells after treatment with rapamycin (0, 1, 10, 20 and 100 μM). Cell survival was evaluated by the clonogenic assay using crystal violet staining. **(B)** Histogram showing the percentage of absorbance at 470 nm. (100% represents the absorbance at 470 nm for untreated cells. (n = 3). ***p < 0.0005.

As shown in **Figure 2**, rapamycin inhibits colony formation in a dose-dependent manner compared with controls, especially in oral cancer cells. No effect was seen in normal cells for the low concentrations (≤ 20 µM), only at the high concentration of rapamycin (100 μM) (**Figures 2A**, **B**). These results obtained with the clonogenic assay were consistent with MTT and LDH assays. For the rest of the study, we used 10 and 20 μM as an average of the IC_50_. With these two concentrations, we next investigated whether the inhibition of oral cancer cell growth/survival was accompanied by induction of their apoptosis through rapamycin treatment.

### Rapamycin promotes Ca9-22 apoptosis by triggering the intrinsic pathway

**Figure 3:**
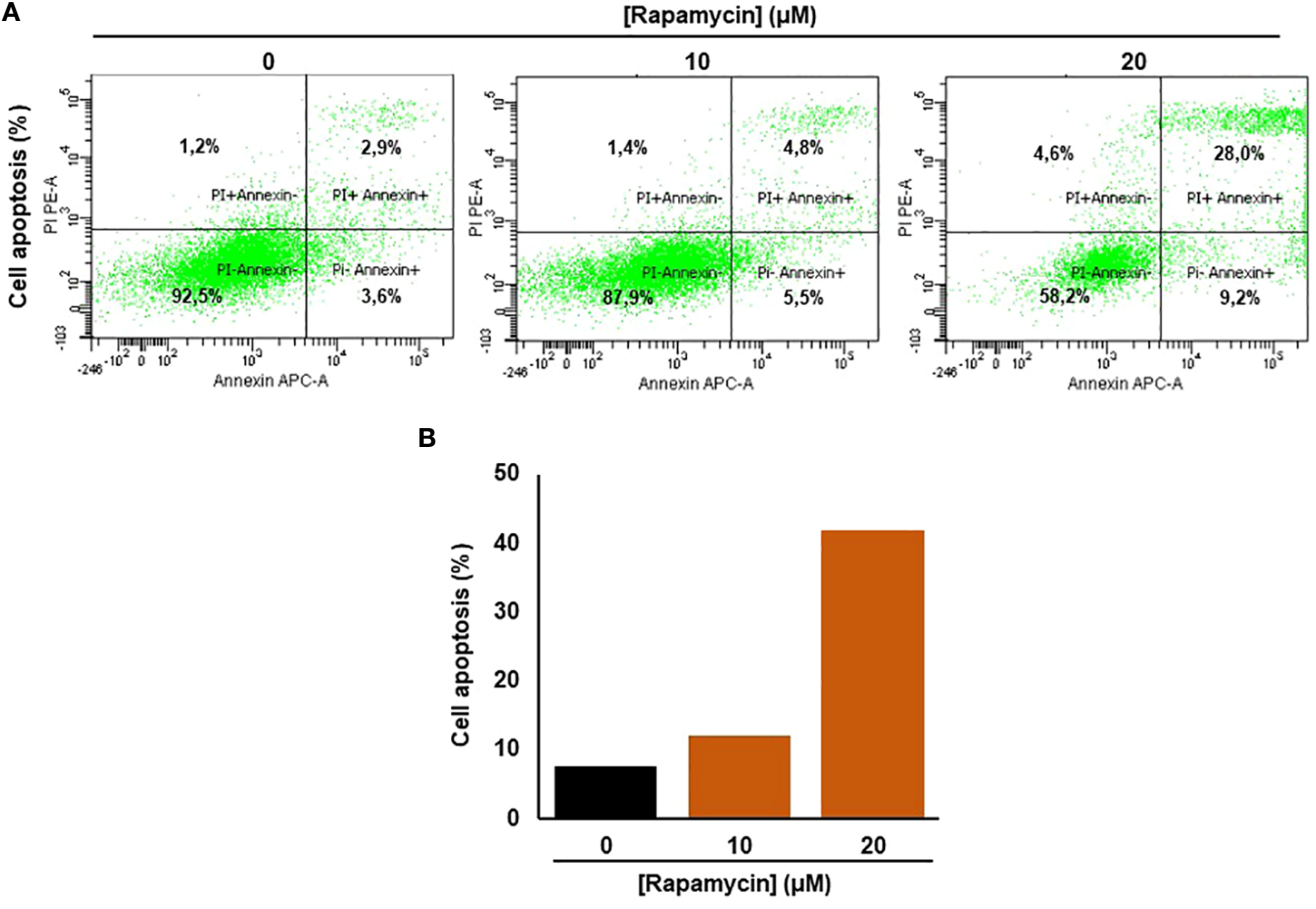
Rapamycin promotes Ca9-22 apoptosis. **(A)** Flow cytometry assay using Annexin/Pi. To measure oral cancer cell apoptotic/cell death after different rapamycin treatments (10 and 20 μM). **(B)** The percentage of cell apoptosis obtained from four individual experiments.

As shown in **Figure 3A**, the stimulation of cancer cells with 10 and 20 μM of rapamycin was causing an increase in cell death and a decrease in living cells. The percentage of the apoptosis cells in untreated cells was 13%, representing necrotic and early and late apoptosis cells. When Ca9-22 cells were exposed to 20 μM of rapamycin, the level of apoptotic cells increased to 41.8% (**Figure 3**). To further confirm the effect of rapamycin on cell apoptosis in more detail, we investigated the effects of 20 µM rapamycin on inactive and cleaved caspase-3 and -9. **Figure 10** shows a decrease in the expression of procaspase 3 and 9 with an increasing concentration of rapamycin. Also, the cleaved forms of caspase-3 and -9 increased following Ca9-22 treatment with rapamycin. Overall, rapamycin promotes cancer cell death through caspases-3 and -9 signaling pathways.

### Rapamycin induces autophagy in Ca9-22 cells

**Figure 4:**
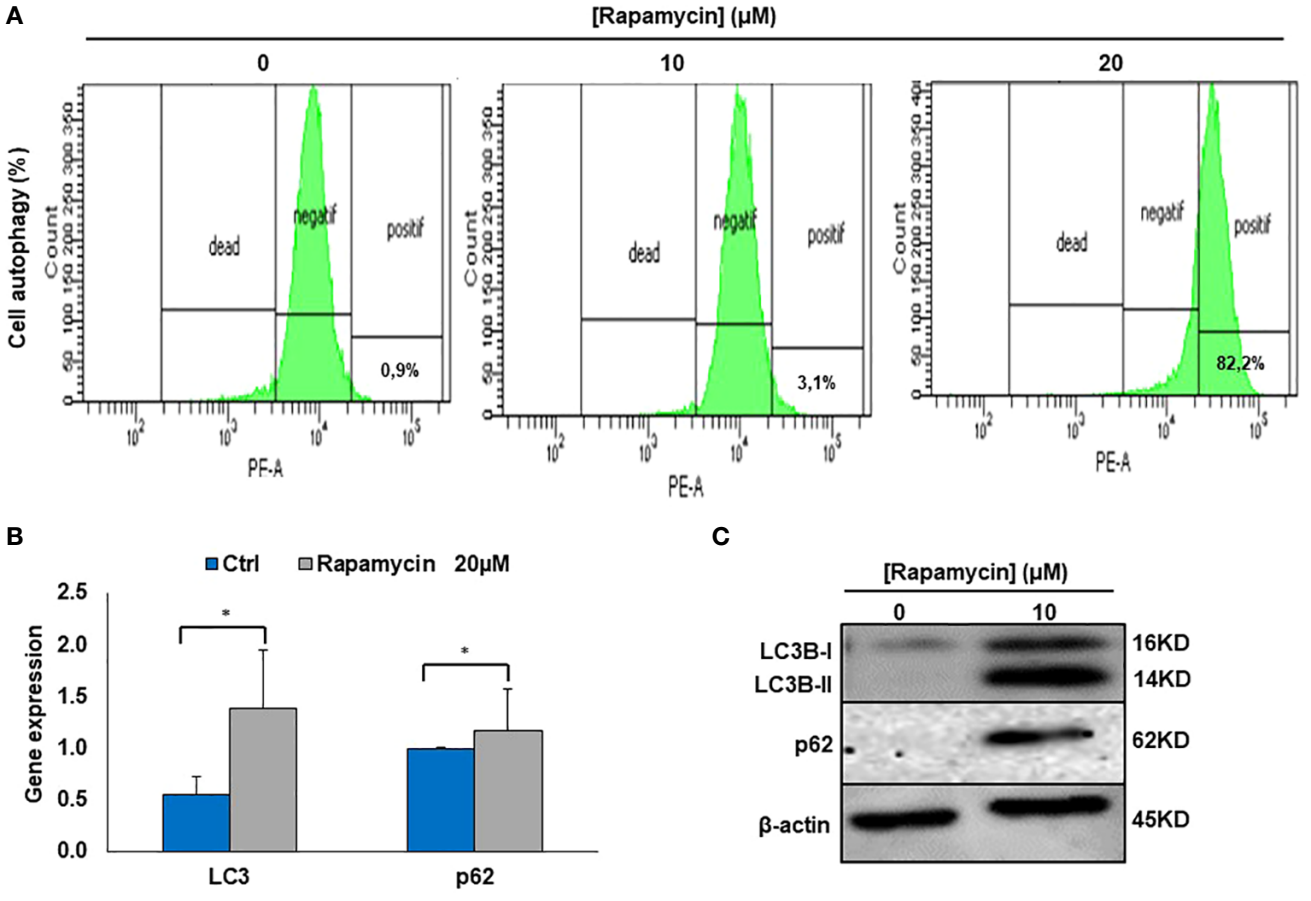
Rapamycin induces autophagy in Ca9-22 cells. **(A)** Flow cytometry analysis (n = 4). To evaluate the effect of rapamycin on autophagy in Ca9-22 cells, the percentage of cellular autophagy was determined using flow cytometry using the Autophagy Red Assay. After 24h of treatment, Ca9-22 cells were stained with the autophagy probe before analyzing them with the green (FL1) channel of a flow cytometer. The results were expressed as means (% autophagy) and are considered significant when *p < 0.05. (n = 4). **(B)** mRNA level of LC3B and p62 by RT-PCR (n=3). **(C)** Protein level of LC3B and p62 by western blotting (n=3).

It was clearly reported that, mTOR is one of the key autophagy inhibitors and rapamycin is one of the best characterized autophagy inducers, we investigated whether rapamycin-induced cell death occurred *via* induction of autophagy. As shown in **Figure 4A**, rapamycin increased the percentage of Ca9-22 cells undergoing autophagy. However, that at basal level was 0.9%. Rapamycin treatment for 24 h significantly increased the percentage of Ca9-22 cells autophagic death from 0.9% with the control to 3.1% with 10 μM, and 82.2% with 20 μM. These results were confirmed by the study of *LC3* and *p62* gene expression study **Figure 4B**. As shown in **Figure 4C**, rapamycin at 20 μM caused an increase in the expression of both genes *LC3B-II* and *p62*. Thus, rapamycin is a promotor of cancer cell autophagy. The autophagosome accumulation induced by rapamycin measured by flux cytometry is probably linked to an increase in autophagy due to increased autophagosome formation. In addition, **Figure 7B** shows that rapamycin at 20µM increases autophagy to 32.9% in ca9-22 compared to 5.9% in untreated cells. However, a pre-treatment with 50µM of 3-MA decreases the percentage of cell autophagic to 13.7%. This autophagy appears to be associated with oxidative stress. In addition, a pre-treatment with 10 mM of NAC also decreases the percentage of cell autophagic from 32.9% in cells treated with 20 µM of rapamycin to 7.6% when the cells were treated by NAC and rapamycin (**Figure 7B**).

### Rapamycin induces oral cancer mitochondrial oxidative stress

**Figure 5:**
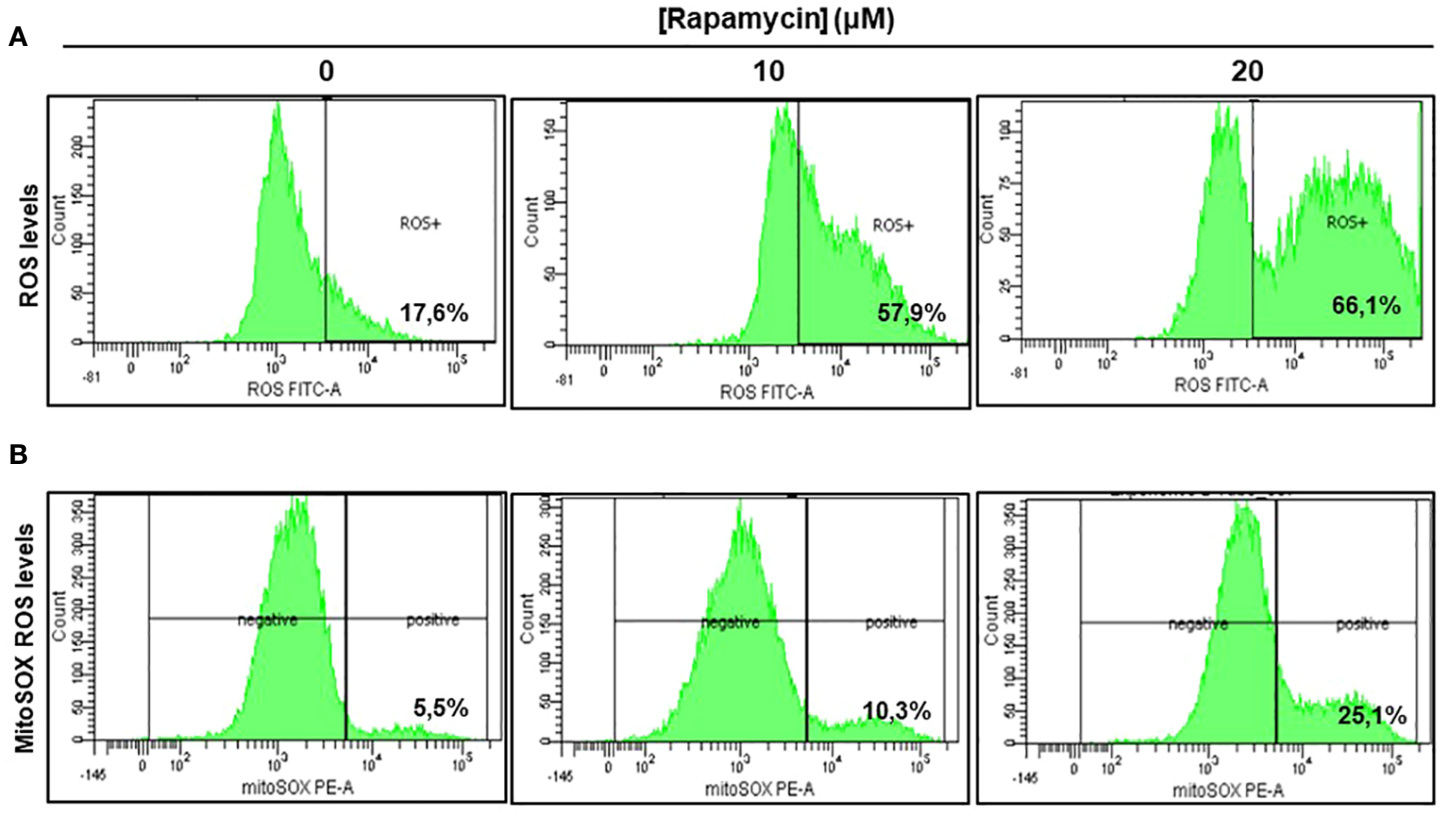
Rapamycin induces oral cancer mitochondrial oxidative stress. **(A)** Rapamycin induces ROS expression in oral cancer cells. ROS levels were measured by flow cytometry using ROS marker protocol. After stimulation with rapamycin at (0, 10 and 20 μM) for 24h, cells were exposed to ROS Green working solution before analyzing them by flow cytometry at 488 nm using BD LSR II or BD FACSCanto II cytometer (BD Bioscience). The percentage of positive cells was calculated in living cells with FACSDiva Software v. 6.1.3. This experiment was repeated four times. **(B)** Measurement of mitochondrial superoxide. The generation of mitochondrial reactive oxygen species (ROS) in rapamycin-treated Ca9-22 cells for 24h was also assessed by flow cytometry using MitoSOX staining, highly selective to detect superoxide in mitochondria of living cells. The results were expressed in percentages of MitoSOX-positive cells (n = 4).

As shown in **Figure 5A** there was a significant induction of ROS in Ca9-22 cells being treated with rapamycin compared to cells untreated. The percentage of ROS increased to 57.9% and 66.1% respectively when the cells were treated with 10 and 20 μM of rapamycin compared to 17.6% in controls (with the vehicle). The generation of mitochondrial ROS in rapamycin-treated Ca9-22 cells for 24h was also assessed by flow cytometry using MitoSOX staining. The percentage of MitoSOX-positive cells also increased with rapamycin concentration. **Figure 5B** shows an increase from 5.5% in untreated cells to 10.3% with 10 μM, and 25.1% with 20 μM of rapamycin. Overall, these results suggest that rapamycin-mediated oral cancer cell apoptosis by mitochondrial-derived ROS production, which occurred upstream of the mitochondrial apoptosis.

### Rapamycin induces oral cancer cell death through oxidative stress and cell autophagy

**Figure 6:**
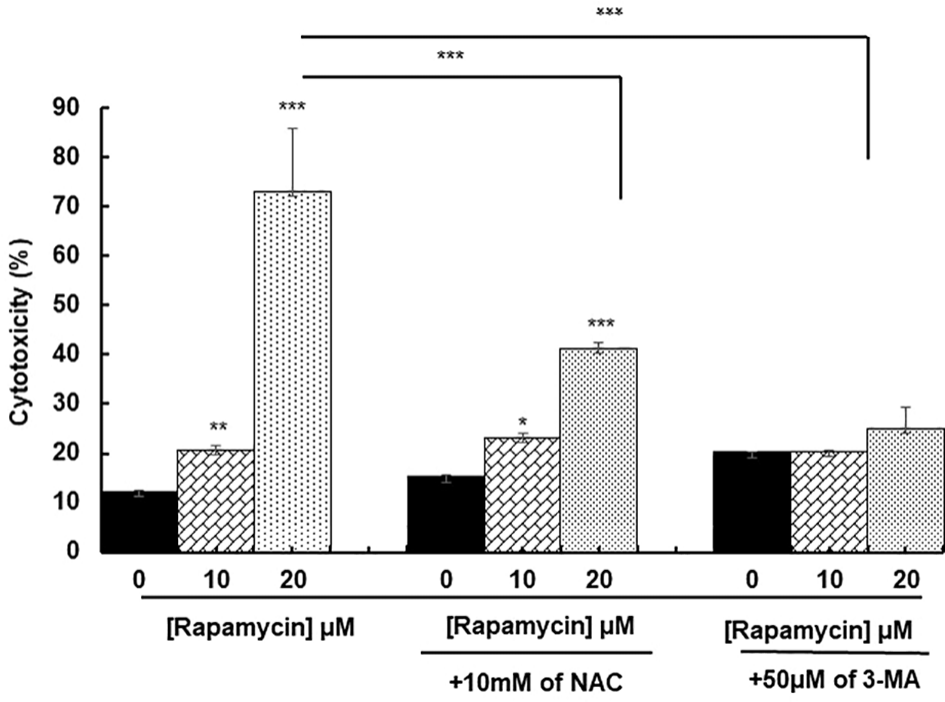
Rapamycin induces oral cancer cell death through oxidative stress and autophagy. Ca9-22 cells were firstly pretreated with or without 10 mM of NAC or 50 µM of 3-MA for 60 min, and then stimulated or not by 10 and 20 µM of rapamycin. After 24 h of rapamycin treatment, LDH assay was performed, and the toxicity rate was calculated with the use of Triton as 100% in cytotoxicity (n = 3). *p < 0.05, **p < 0.005 or ***p < 0.0005.

**Figure 7:**
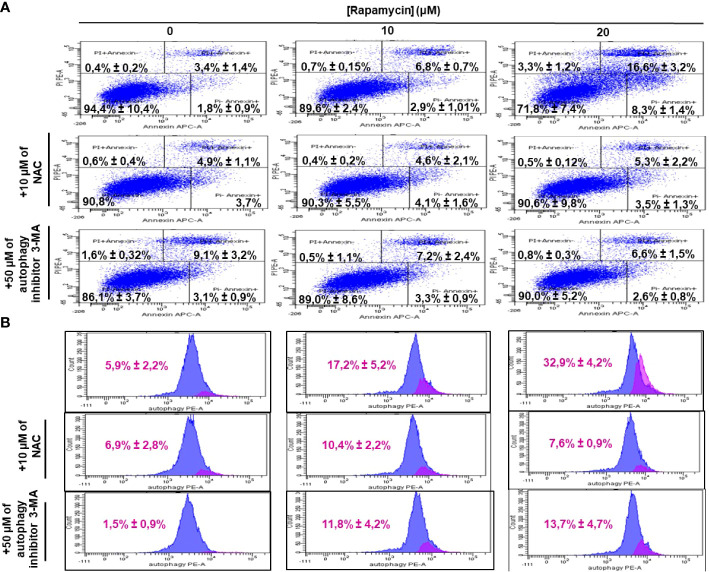
Inhibition of oxidative stress and autophagy reverses rapamycin-induced apoptosis. Ca9-22 cells were firstly pretreated with or without 10 mM of NAC or 50 µM of 3-MA for 60 min, and then stimulated or not by 10 and 20 µM of rapamycin. After 24 h of rapamycin treatment, Annexin V/PI assay for evaluating the percentage of apoptotic cells was performed in **(A)** and the percentage of autophagic cells was evaluated in **(B)** (n = 3).

ROS generation has been recognized to induce stress-mediated cell death in a variety of cancer types. Our hypothesis was that rapamycin triggers cell death *via* ROS production. As shown in **Figures 6** and **7**, the toxicity rate significantly increased with rapamycin concentration. The inhibition of oxidative stress by 10 mM of N-acetylcysteine (NAC) dramatically reversed the effect of rapamycin. In addition, the percentage of toxicity cutoff in cells decreased from 73.08% ± 12.57% with 20 µM of rapamycin to 41.18% ± 1.18% with the same concentration but having a pretreatment with 10 mM of NAC for 60 min. Similar results were observed for autophagy inhibition, a pretreatment of Ca9-22 cells was carried out with 50 µM of 3-methyladenine (3-MA); this inhibitor decreased the above percentage from 73.08% ± 12.57% with 20 µM of rapamycin to 24.96% ± 4.51% when cells were pretreated with 3-methyladenine for 60 min and then stimulated by 20 µM of rapamycin (**Figure 6**). Inhibition of oxidative stress or autophagy also reversed rapamycin-induced apoptosis. As shown in **Figure 7**, the percentage of apoptotic cells increased with rapamycin concentrations, particularly with 20 µM (28.2%). This induction of apoptosis was inhibited up to 9.3% and 10% when cells were pretreated respectively with NAC and 3-MA.

### Rapamycin-induced DNA damage through g-H2AX- expression

**Figure 8:**
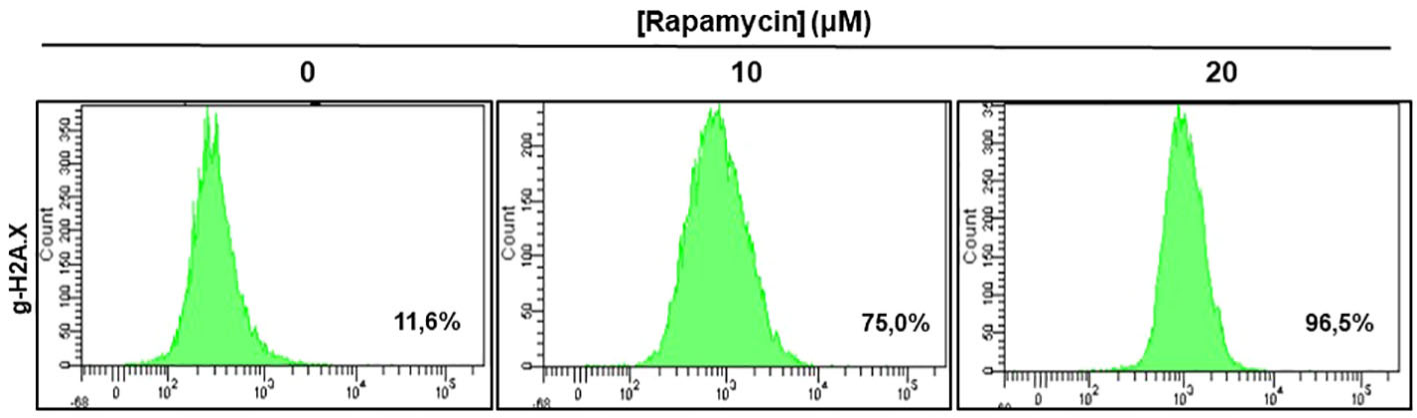
Rapamycin-induced DNA damage in Ca9-22 cells: The DNA damage was determined by g-H2AX-based flow cytometry. Ca9-22 cells were treated with 10 and 20 μM of rapamycin for 24h and the damage was evaluated by flow cytometry using g-H2AX antibody (n = 3).

**Figure 8** shows the effect of rapamycin on the expression of g-H2AX by Ca9-22. The percentage of g-H2AX-positive cells increased significantly following exposure of oral cancer cells to rapamycin. Indeed, the percentage of g-H2AX-positive ranged from 11.6% in control cells to 75% with 10 μM, to 96% with 20 μM of rapamycin (**Figure 8**).

### Rapamycin suppresses cell migration and invasion in Ca9-22 cells

**Figure 9:**
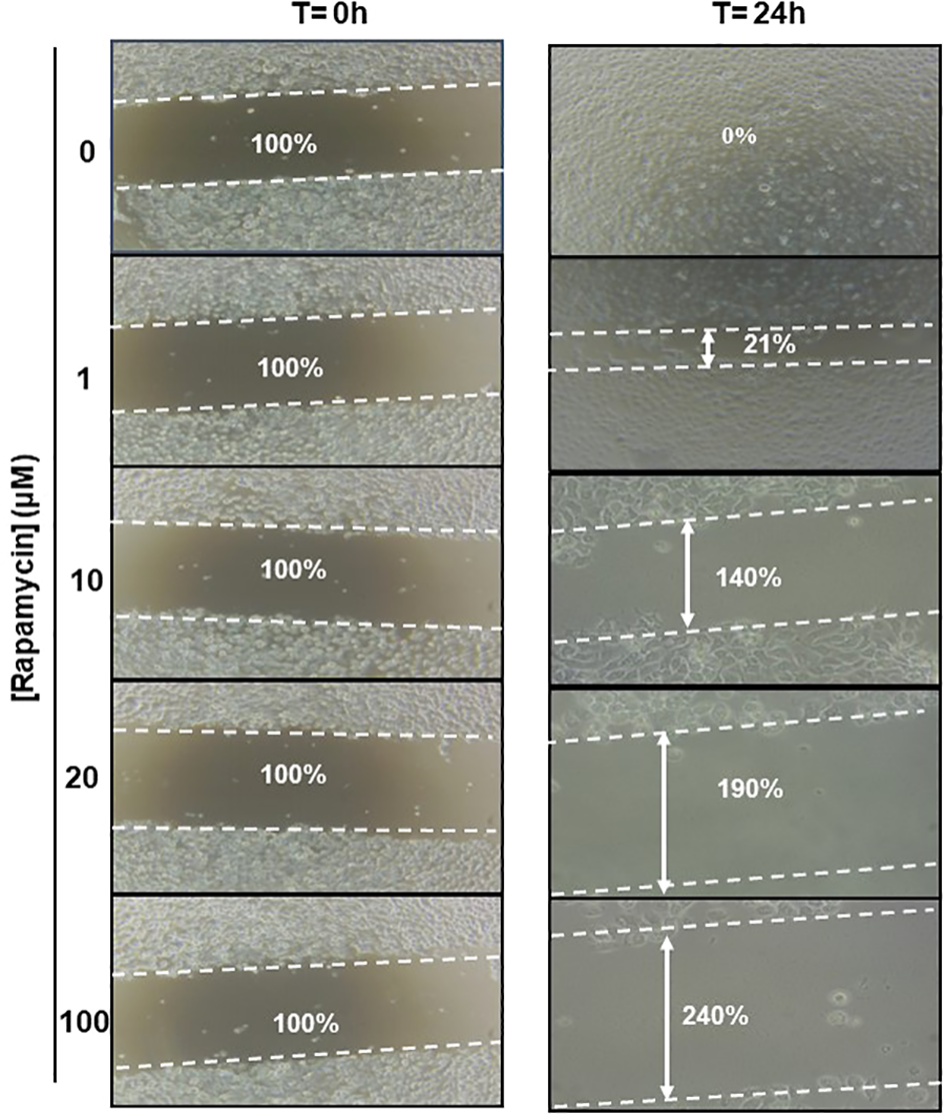
Rapamycin suppresses cell migration and invasion in Ca9-22 cells. Wound-healing assay. The effect of rapamycin on cell migration was determined by the scratch method. Non-stimulated cells were compared to Ca9-22 stimulated with concentrations of 1, 10, 20 and 100 μM of rapamycin. The cell migration was analyzed by image processing software that was able to measure the distance between opposite edges of the scratch at each time point. Each well was then compared, based on their percentage of closure.

The effect of rapamycin on cell migration was determined by the scratch method. Non-stimulated cells were compared to Ca9-22 stimulated with concentrations of 1, 10, 20, and 100 μM of rapamycin. As shown in **Figure 9**. the wound-healing assay demonstrated that rapamycin inhibited the Ca9-22 cell migration and invasion. Non-stimulated cells migrated over the scratch made. However, migration of rapamycin-treated cells was inhibited in a dose-dependent manner.

### Rapamycin inhibits MAPK, NF-κB and β-catenin signaling pathways and activates caspase pathways

**Figure 10:**
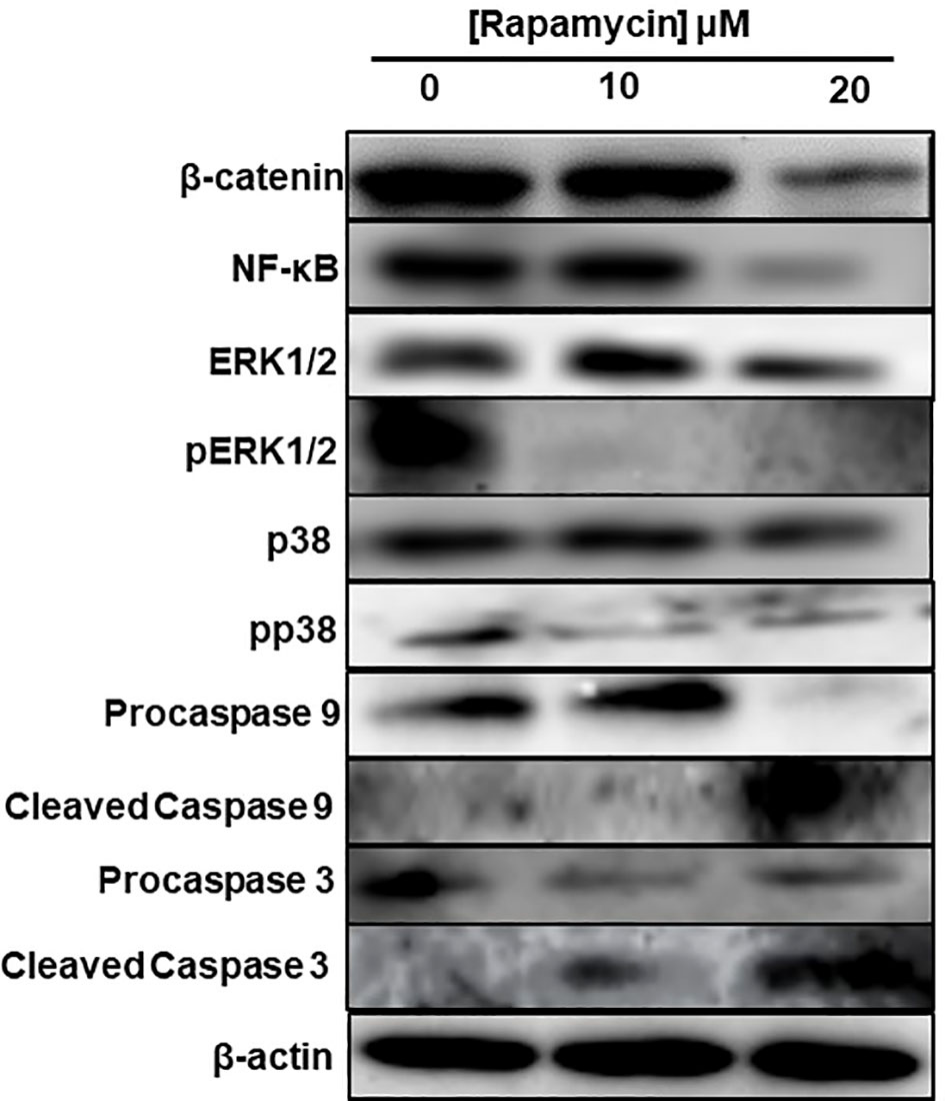
Rapamycin inhibits MAPK, Wnt/β-catenin signaling pathways and activates caspase pathways. An amount of each protein varying between 20 μg and 60 μg was needed for Western blotting analysis. We used specific primary antibodies to β-catenin, Wnt/beta catenin, MAP kinase (ERK1/2 and p38) and that of caspases. (n = 3).

To investigate what signaling pathways related to cancer progression were targeted by rapamycin in oral cancer cells, we analyzed β-catenin, NF-κB, MAP kinase (ERK1/2 and p38) and that of caspases. As shown in **Figure 10**, there is a decrease in the expression of β-catenin, a pathway involved in cell adhesion as well as for NF-κB a pathway implicated in inflammation. Also, rapamycin decreased the activation of ERK1/2 and p38 pathways but was not affecting the total ERK1/2 and p38. Unlike proliferation pathways, rapamycin is thus activating two key pathways involved in apoptosis, particularly cleaved caspase-3 and cleaved caspase-9 pathways, confirming the results presented in (**Figure 10**).

## Discussion

The goal of the current study was to investigate the inhibitory role of rapamycin on oral cancer progression and its potential use as an alternative or complementary agent to the conventional cancer treatment. We demonstrated that a low dosage of rapamycin shows a selective effect on cell growth/survival in oral cancer, with the cell cycle likely blocked by CDKs inhibitors, being crucial for the orderly initiation and progression of the cell-division cycle. It involves modulating the synthesis of CDK inhibitors, such as p21, p15 and p27, found in quiescent cells at high levels, but they are downregulated by mitogenic stimulation (20, 21), and by inhibition of cyclin D1 expression, both are rate-limiting for entry into S phase. Rapamycin increases levels of CDK inhibitors such as p27^Kip1^. These results are consistent with the data published by other groups reporting that rapamycin has multiple biological functions, including anticancer activity (22, 23), and it is considered as one of the key potential chemopreventive agents in therapy causing suppression, or inversion of carcinogenesis (24, 25). Several studies have reported that rapamycin alone or in combination with chemotherapeutic agents inhibits the proliferation of various tumor cells (26, 27). It also inhibits the cell cycle progression, particularly the G1/S transition by targeting mTOR, and cell growth effectors S6K1, 4E-BP1, and cyclin D1 (28, 29).

Importantly, another anticancer property of rapamycin is its ability to promote cancer cell apoptosis. Indeed, we demonstrated that rapamycin promoted Ca9-22 cell apoptosis through the activation of caspase-9 and -3. The effect rapamycin may induce oral cancer cell apoptosis directly by suppressing 4E-BP1 phosphorylation through mTORC1 and indirectly by inactivating eIF4E. Therefore, these data indicate that inhibition of mTOR by rapamycin is closely linked to cell growth and apoptotic processes due to the inactivation of the mTOR pathway and its downstream target genes. These results are consistent with those previously published studies (30–32). Recently, Jun Yao etal. (31) have reported that 0.2 µM and 0.4 µM of rapamycin increased the number of apoptotic cells and the cell cycle of retinoblastoma cells was basically stopped in S phase and consequently, the expression levels of Bcl-2, PI3K and AKT declined with rapamycin stimulation at 0.2 µM and 0.4 µM (31). On the other hand, we have shown that rapamycin induces significantly oral cancer cells undergoing autophagy by increasing the LC3 and p62 expression. Data in concordance with these same recent studies, which reported that targeting the induction of autophagy may be an excellent emerging strategy for cancer therapy (33); (34). In addition, it was demonstrated that rapamycin inhibits cell proliferation and induces autophagy in human neuroblastoma cell lines by suppressing the mTOR signaling pathway through increasing gene expression of LC3-II/LC3-I and Beclin (35). We strongly believe that the induction of autophagy by rapamycin in oral cancer cells is due to its ability to specifically, inhibit mTORC1. It was clearly reported that rapamycin is considered as an allosteric mTORC1 inhibitor (36–38). However, these same research groups reported that mTORC1 is inhibiting the autophagy-initiating kinase UNC-5 like autophagy activating kinase 1 (ULK1) complex by phosphorylation of complex components, including autophagy-related 13 (ATG13) and ULK1/2 genes (39–42).

Rapamycin could have anti-tumor activity through mitochondrial-derived ROS and DNA damage. We showed that rapamycin increased the accumulation of ROS in cancer cells, as previously reported (22, 43–46). Moreover, we have demonstrated that rapamycin dramatically induces DNA damage in oral cancer cells as measured by histone H2AX phosphorylation, one of the highly sensitive and general markers induced by chemotherapy (47). The consequent accumulation of damaged DNA in Ca9-22 cells is probably due to the ability of rapamycin to promote oral cancer cell cycle disruption, which is closely associated with further increased replication stress (42). However, it is known that replication stress is considered as a key cause of DNA damage and high genomic instability, two main features of cancer cells. Oral cancers are characterized by the invasion/migration capacity of malignant cells, often accompanied by disruption of the extracellular matrix (ECM). Our results show that rapamycin suppressed cell migration and invasion in Ca9-22 cells at the epithelial-to-mesenchymal transition (vimentin and E-cadherin) (data not shown). The same observations were found in our previous studies using a natural product (16), or with the analog of curcumin (14) on oral cancer cells. Song et al. (48) have reported that rapamycin treatment leads to growth arrest and inhibition of invasion in human chondrosarcoma cells (48). Recently, Sahu et al. (49) showed that bladder cancer invasion was closely mediated by mammalian target of rapamycin (49). The rapamycin inhibitory effect of cancer cell proliferation could be related to the expression level of proteolytic enzymes such as plasminogen activator (PAs) and matrix metalloproteinases (MMPs) known to be key factors involved in the tumor cell invasion and metastasis (12). MMPs, like MMP-2 and MMP-9, play a role in ECM degradation and are highly expressed in carcinomas to promote tumor angiogenesis and consequent cancer cell invasion and metastases. Inhibiting MMP represents a real-world new therapeutic strategy for several cancer treatments, thus various MMP inhibitors are currently being assessed for clinical applications. In this study, Ca9-22 cells treated with rapamycin were not enabling the activation of MAP kinases (in particular, ERK1/2) and NF-κB, which are involved in the transcriptional regulation of proteolytic enzymes. The overactivation of ERK1/2 has been reported to be involved in cancer progression (50–52). In addition, NF-κB pathway is shown to induce inflammation and cancer cell invasion *via* increasing MMPs. In fact, several evidences support that rapamycin inhibits NF-κB (53). These results suggest that the inhibitory effect of rapamycin on the motility of Ca9-22 cells might be associated with its ability to inhibit the activation of MAP kinases, beta-catenin, and NF-κB.

**Figure 11:**
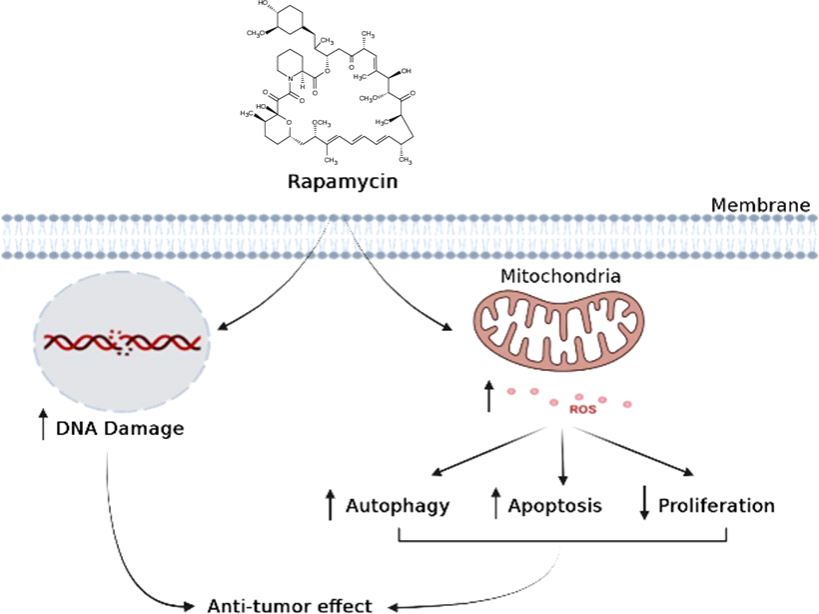
Schematic model proposed for the mechanism of action of rapamycin in oral cancer cells.

In conclusion, when cancer cells are stimulated with increasing concentrations of rapamycin, it is possible to observe an increase in cell death and autophagy, as well as an inhibition of the cell proliferation, colony formation, cell adhesion, inflammation, and cell migration. As shown in (**Figure 11**), rapamycin has an anti-cancer effect by inducing DNA damage to Ca9-22 cells and inducing their oxidative stress, which, in turn, induces Ca9-22 cell autophagy and apoptosis and inhibits their proliferation (**Figure 11**). These findings show that rapamycin is a potential agent that could be used in the treatment of oral cancer.

## Data availability statement

The original contributions presented in the study are included in the article/supplementary material. Further inquiries can be directed to the corresponding author.

## Author contributions

AS, conceptualization, conducted experiments, and writing - original draft. SP, CC, and IZ, conducted Western blotting and flux cytometry experiments. MR, critical revision of the article, supervision, review, and editing. All authors contributed to the article and approved the submitted version.

## Funding

This work was supported by a grant (FO131038) from the “Fonds Émile-Beaulieu” at Laval University and by grant (FO130688) from Network for Canadian Oral Health and Research.

## Acknowledgments

The authors are thankful to RSBO (Réseau de Recherche en Santé Buccodentaire et Osseuse) for providing financial support.

## Conflict of interest

The authors declare that the research was conducted in the absence of any commercial or financial relationships that could be construed as a potential conflict of interest.

## Publisher’s note

All claims expressed in this article are solely those of the authors and do not necessarily represent those of their affiliated organizations, or those of the publisher, the editors and the reviewers. Any product that may be evaluated in this article, or claim that may be made by its manufacturer, is not guaranteed or endorsed by the publisher.
